# Baculovirus Induced Transcripts in Hemocytes from the Larvae of *Heliothis virescens*

**DOI:** 10.3390/v3112047

**Published:** 2011-10-28

**Authors:** Jonathan E. Breitenbach, Kent S. Shelby, Holly J.R. Popham

**Affiliations:** Biological Control of Insects Research Laboratory, Agricultural Research Service, USDA, 1503 S. Providence Road, Columbia, MO 65203, USA; E-Mails: Jonathan.Breitenbach@ars.usda.gov (J.E.B.); Kent.Shelby@ars.usda.gov (K.S.S.)

**Keywords:** baculovirus, lepidopteran, transcription, RNA-seq

## Abstract

Using RNA-seq digital difference expression profiling methods, we have assessed the gene expression profiles of hemocytes harvested from *Heliothis virescens* that were challenged with *Helicoverpa zea* single nucleopolyhedrovirus (HzSNPV). A reference transcriptome of hemocyte-expressed transcripts was assembled from 202 million 42-base tags by combining the sequence data of all samples, and the assembled sequences were then subject to BLASTx analysis to determine gene identities. We used the fully sequenced HzSNPV reference genome to align 477,264 Illumina sequence tags from infected hemocytes in order to document expression of HzSNPV genes at early points during infection. A comparison of expression profiles of control insects to those lethally infected with HzSNPV revealed differential expression of key cellular stress response genes and genes involved in lipid metabolism. Transcriptional regulation of specific insect hormones in baculovirus-infected insects was also altered. A number of transcripts bearing homology to retroviral elements that were detected add to a growing body of evidence for extensive invasion of errantiviruses into the insect genome. Using this method, we completed the first and most comprehensive gene expression survey of both baculoviral infection and host immune defense in lepidopteran larvae.

## 1. Introduction

Baculoviruses are insect-specific pathogens that have been utilized as both tools for recombinant protein expression and biological agents targeting agriculturally significant pests, most importantly within the order Lepidoptera [1–4]. *Helicoverpa zea* single nucleopolyhedrovirus (HzSNPV) infects several species of noctuid moths with outcomes ranging from a low rate of infection to 100% mortality, depending on the host species [5–7]. This range of outcomes points to the importance of the host response to viral infection, about which little is understood at the molecular level, within the infected animal. Methodologies that employ DNA microarray and gene chip have been utilized to probe pathogen-host interactions at the transcriptional level to shed light on factors that determine the outcome of infection, but these methods rely on existing consensus DNA sequence information for a given species [8–10]. While such studies are possible in genetically well-defined experimental models, this limitation has restricted genetic and transcriptional analysis of less intensively studied hosts and their interactions with relevant pathogens [11–13]. Indeed, the bulk of insect host response to viral infection has been related to the dipteran response to infection with RNA viruses, chiefly *Drosophila melanogaster* [14–17] and mosquitoes [18–20].

RNA-seq studies of host gene expression allow quantitation of gene expression across an entire genome in response to infection [21,22]. Examination of host gene expression in response to baculovirus infection has been the subject of relatively few studies [23]. Based on our prior observations, and those of others, we hypothesized that baculovirus infection of a permissive species would result in substantial changes in the transcriptional profile of the host. To address this hypothesis in a biologically relevant *in vivo* context, both baculovirus and host gene expression were documented simultaneously in the same tissues of infected *Heliothis virescens* larvae by an RNA-seq approach using an Illumina GAII sequencing instrument. Illumina is a sequencing method that generates up to a terabase of sequence information as short (approximately 40 bases) sequence reads from input samples of nucleic acids [23]. By barcoding the nucleic acids of each sample, multiple treatments, time points, controls and different species were crowded onto a single lane, allowing a fine grained understanding of the regulation of thousands of genes simultaneously. Illumina RNA-seq has been used to examine differential gene expression during whitefly infection by tomato yellow leaf curl china virus, and the corn planthopper subsequent to infection with Maize mosaic rhabdovirus [24,25].

After assessing sample quality and screening out contaminant rRNA and tRNA, we constructed a *de novo* transcriptome of approximately 100,000 *Heliothis virescens* hemocyte transcripts and subsequently aligned sequence tags from control and HzSNPV-infected hemocyte treatments to this reference transcriptome. Using a digital expression profiling approach comparing control and infected samples, we observed overall trends in important cellular processes closely related to the natural history of infection, and expanded on previous studies that focused more narrowly on the differential expression of specific host genes consequent to infection [26]. Taken together, this study surveys the comparative transcriptional state of infected larvae to reveal pathogen-specific alterations in gene expression and simultaneously represents the largest deposition of *H. virescens* mRNA sequence information in a publicly-accessible database to date.

## 2. Results

### 2.1. Transcriptome Generation and Assembly

The treatment and enumeration of the Illumina sequence output is summarized in Table 1. A total of 202 M 32–42 nt base tags from each of the three treatments and control lanes (Table 1, Total Seqs) were pooled, cleaned, and screened (Table 1, After Decontamination) for tRNA and rRNA sequences and assembled as described in the Experimental Section. A reference *de novo* transcriptome assembly of 103,879 sequences was thus generated from this output of hemocytes from HzSNPV infected, bacterial- and fungal-stimulated larval hemocytes. Approximately 31 million of the control reads aligned to this reference assembly, and of these almost 20 million aligned uniquely. While mRNA derived from hemocytes of bacterial and fungal septic puncture treated insects were utilized in constructing the *de novo* transcriptome assembly, the subsequent gene regulation data for those treatments are outside the scope of this manuscript and will be reported elsewhere.

### 2.2. Illumina Reads Matching the HzSNPV Genome

**Figure 1.**
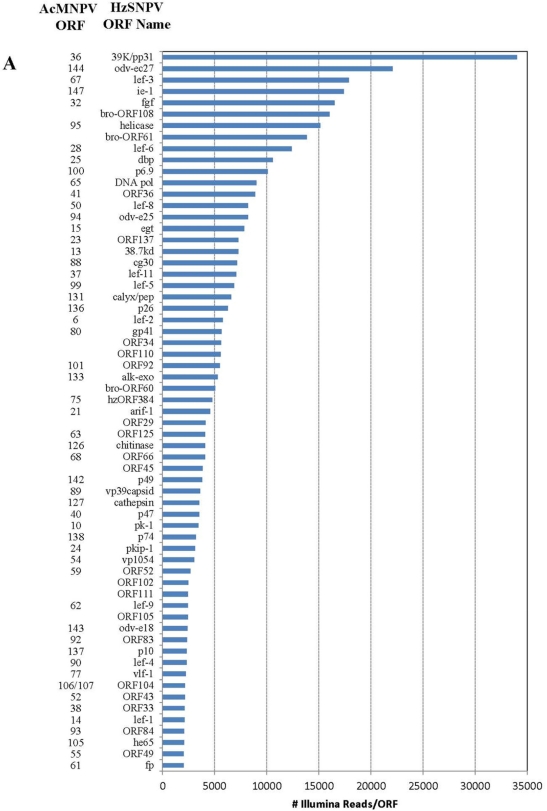

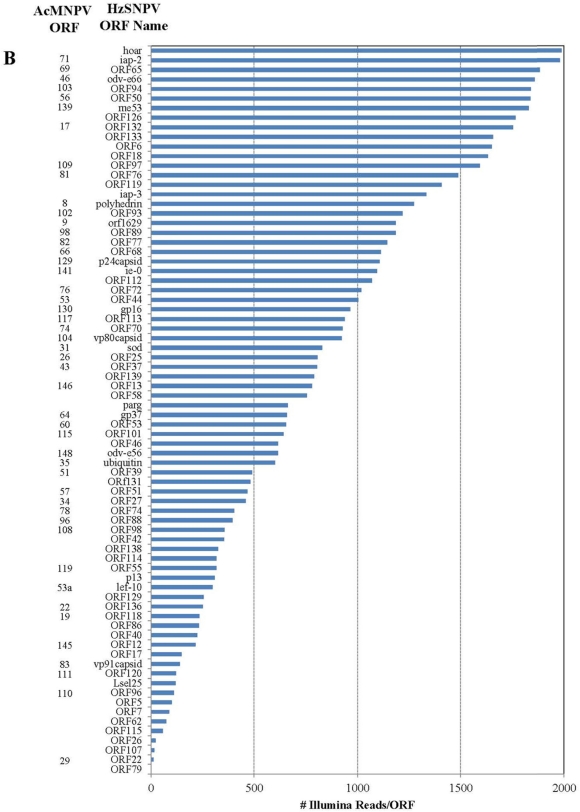
Number of individual Illumina reads that aligned completely within each *Helicoverpa zea* single nucleopolyhedrovirus (HzSNPV) open reading **f**rame (ORF). The corresponding *Autographa californica* multiple nucleopolyhedrovirus (AcMNPV) ORFs are listed to the left of the HzSNPV ORF name. Reads are listed in decreasing order of Illumina read matches. Panel (**A**) Matches >2000 and (**B**) matches <2000.

Of the 202 million reads used to assemble the Illumina library, 477,264 aligned with the HzSNPV genome (approximately 0.26%) [27]. The majority of the reads aligned uniquely within one of the 139 HzSNPV open reading frames (ORF) (Figures 1A,B). The ORF with the highest number of matching reads was ORF31 38K/pp31 (34,047 matches) followed by ORF11 odv-ec27 (22,096 matches) which correspond to the *Autographa californica* multiple nucleopolyhedrovirus (AcMNPV) ORFs 36 and 144, respectively (Figure 1A) [28]. Many of the ORFs with the lowest number of matching reads were ORFS that are to date specific to HzSNPV and/or *Helicoverpa armigera* SNPV (Figure 1B) [27]. Only 4064 Illumina reads aligned to more than one ORF (Table 2), and these reads were spread between 14 ORFs. The reads that aligned to more than one ORF did so because of overlapping areas between two ORFs. The difference in number of hits refers to the difference between the number of reads that are unique to the ORF and those that match to the area of two overlapped ORFs.

Four ORFS have previously been found that are unique to HzSNPV (ORF26, ORF42, ORF62, and ORf79) [27]. ORF79 had two Illumina read matches which was the lowest number of reads for any ORF and suggests that it is not a functional ORF. ORF26 had 29 hits but seven of these potentially overlapped ORF27. ORF62 had 74 hits, which was greater than the number of matches for ORFs 22, 107 and 15. ORF42 had 354 matching reads, which was more than *lef-10* (298 hits) and encodes for a similar size protein, adding to the likelihood that it is a functional ORF.

### 2.3. HSP70/Small HSPs

In hemocytes isolated from insects infected with baculovirus, transcription of heat-shock protein 70 (*hsp70*) genes was up-regulated 70- and 176-fold over control levels, and two transcripts comprised of 1763 and 1452 nt were assembled that bore homology to *hsp70* of *Spodoptera exigua* and *Helicoverpa zea*, representing potentially different isoforms of this gene (Table 3). Four small HSP transcripts encoding HSPs of 19 to 21 kiloDaltons (kD) were identified as upregulated between 16- and approximately 21-fold (Table 3).

### 2.4. Cellular Adhesion/Immunity Genes

Baculovirus infection suppressed transcription of cell adhesion molecules *dystroglycan*, and *fasciclin-1* to 0.3 and 0.02 (three- and 50-fold) relative to control levels, respectively, whereas a gene identified as EGF-containing fibulin-like extracellular matrix protein 2 was induced seven-fold over control (Table 3). Transcription of the immunity-related signal transducing adapter molecule 2 was suppressed approximately 4.5-fold, while *cecropin D* was upregulated by 12-fold in response to viral infection. Larval cuticle protein 2 was also up-regulated following baculovirus infection.

### 2.5. Endocrine Transcripts

A gene identified as probable nuclear hormone receptor *HR3* was upregulated nearly eight-fold in response to baculovirus infection (Table 4). Hormone receptor 3C, juvenile hormone epoxide hydrolase, and nuclear hormone receptor *FTZ-H1F* were upregulated between approximately three- to four-fold. Transcription of basic juvenile hormone-suppressible protein 1 and 2 were suppressed in the presence of virus by 40- and 200-fold, respectively; juvenile hormone diol kinase was also down-regulated following baculovirus infection.

### 2.6. Metabolic Regulation

mRNA sequences bearing significant homology to stearoyl-CoA desaturase, fatty acid reductase, and fatty acid synthase genes were upregulated by baculovirus infection (Table 4). A total of sixteen genes related to lipid metabolism were induced by viral infection with magnitudes of induction in response to baculovirus that ranged from 3- to 237-fold. Four genes encoding enzymes involved in this pathway were down-regulated in response to baculovirus infection; among these was fatty acid binding protein 3, which functions to bind to unsaturated fatty acids. Genes identified as *p260*/*p270*, thought to be *fatty acid synthase* homologs, were upregulated in response to HzSNPV infection: *p260* by more than 400-fold, and *p270* by 51-fold. Related to this metabolic pathway, the transcription of genes encoding storage proteins *arylphorin*, *p82* (riboflavin-binding hexamer), and *hexamerine* was suppressed between 20- and 100-fold.

### 2.7. Errantivirus

More than a hundred genes that were differentially regulated in response to baculovirus infection were identified as homologs of endogenous retroviral elements or retrotransposon related genes. In insects, these are termed errantiviruses [29]. Because of the large number of these transcripts, an arbitrary reporting requirement of at least 45 sequence detections of a given gene in either the control or virus-infected samples was established. With one exception, the remaining genes were upregulated between 3.3- and 11.25-fold compared to those detected in the control hemocytes (Table 5).

## 3. Experimental Section

### 3.1. Insects, Infection, and RNA Isolation

*Heliothis virescens* eggs were received from the North Carolina State University, Dept. of Entomology Insectary (Raleigh, NC, USA). Larvae were reared individually on an artificial wheat germ based diet (Bio-Serv, Frenchtown, NJ, USA) under standard conditions of 14 h:10 h (L:D) photoperiod, 55% RH, 28 °C [30]. Synchronized early-4th instar larvae (4 hours post molt) were either mock-infected with a control inoculum absent virus or infected with an LC99 dose of HzSNPV *per os* (5 × 10^8^ polyhedra/mL) [30]. To activate the antibacterial immune response early 5th instar larvae were punctured with a tungsten needle dipped into a 1 μg/mL suspension of lipopolysaccharide and peptidoglycan (Sigma Chem. Co., St. Louis, MO, USA) in PBS [31]. The antifungal response was activated by puncture with a tungsten needle dipped into a 1 μg/mL suspension of β-glucan, curdlan and laminarin (Sigma Chem. Co., St. Louis, MO, USA) in PBS. Hemocytes were collected at 12 hpi in order to capture early host-responses to HzSNPV infection. Hemocytes were collected from 30 insects from each treatment according to previously published methods [32]. Larvae were bled through a punctured anterior proleg into ice cold PBS containing a crystal of phenylthiourea to prevent melanization. Hemocytes were pelleted by centrifugation at 5000 × *g* for 4 min, plasma supernatant was removed, and stored at −85 °C for later use. Total RNA was extracted from hemocytes using RNeasy™ kits (Qiagen, Valencia, CA, USA) [33].

### 3.2. Sequence Generation, Assembly and Annotation

Hemocyte RNA pools were submitted to University of Missouri, Bond Life Sciences Center DNA Core for Illumina GAII sequencing. RNA quality of the pooled time points was examined using the Experion Automated Electrophoresis system (Bio-Rad, Hercules, CA, USA). Libraries were constructed according to the standard Illumina RNA-seq protocol (Part# 1004898 Rev. A, rev Sept 08) for the pooled PCR products except for the fragmentation step [34]. First strand and second strand cDNA synthesis was carried out with Invitrogen superscript II reverse transcriptase and random hexamer primers according to the manufacturers protocol. Illumina paired-end adapters were ligated and a gel size-selected ∼300 bp fragments depleted of adapter dimer and large chimeric fragments were subjected to 15 rounds of selective PCR enrichment. The four treatments were run on four separate lanes on an Illumina GAIIx sequencing instrument. To assemble the *de novo* hemocyte transcriptome the sequence data from all lanes was combined yielding over 202 million 42-base long Illumina single end reads which were first cleaned of low quality bases at the 3′ end, and then cleaned for low quality overall using the Fastx Toolkit [35]. The remaining reads were screened for homology to contaminants (predominantly rRNA) to leave 192 million reads of lengths 32 to 42. VELVET was used to assemble the reads into preliminary contigs using a range of word size (K values) from 21 to 41 [36]. OASIS was used to assemble the VELVET output into transcript isoforms of at least 100 bases in length [37]. Those reads that were not assembled under the first round were used as input to a second round of assembly by VELVET and OASIS. The results for each value of the word size were combined and redundant transcripts were removed using Vmatch resulting in 103,879 unique potential transcripts [38]. These sequences were annotated by homology using BLASTx with proteins in the UniProt uniref100 database [39]. About two dozen sequences were identified as chimeric and removed. Functional categories of contig and singleton sequences resulting from this assembly were annotated using a local installation of BLAST2GO [40]. All sequence data discussed in this manuscript have been deposited in NCBI GenBank [41].

### 3.3. Alignment of Sequence Tags with HzSNPV Reference Genome

The cleaned reads from the various samples were aligned to the HzSNPV genome using SOAP [42]. ORFs were defined from reported coordinates at GenBank [27].

### 3.4. Identification of Differentially Regulated Genes

Host gene expression was analyzed in a semi-quantitative fashion by comparison of the control and HzSNPV Illumina lanes, normalizing the number of times sequence tags aligning with an mRNA was detected in the cells of HzSNPV infected larvae to those of controls. We analyzed genes that were shown to be upregulated or down regulated by a factor of three and that had an e-value less than 0.0001, and those findings are presented in Tables 3 through 5.

## 4. Discussion and Conclusions

Previous work has shown that during HzSNPV infection of *H. virescens* larvae, infection of midgut columnar epithelial cells could be detected by 4 hours post infection but remained constant by 24 hours post infection [43,44]. In larvae infected with HzSNPV-hsp70/LacZ using a dose killing 84% of a test group, 40% of the larvae were LacZ positive in either the midgut or midgut and tracheal epithelia [43]. In this study, we applied an RNA-seq approach to examine transcription of hemocytes extracted from *H. virescens* that were experimentally challenged with HzSNPV to capture the early transcriptional profile of the baculovirus genome within the first 12 hours of infection (Figures 1A,B). At 12 hours post ingestion, we expected to capture a time point at which the primary infection of the midgut had begun to progress into the circulating hemocytes, in attempt to identify the host genes that are potentially involved in an immune response to the virus. Surprisingly, we detected transcription of all known HzSNPV ORFs at this time point at varying levels of expression. While this analysis provides some detail of the level to which these genes are transcribed early in infected hemocytes, more infection time points would be necessary to precisely follow gene expression levels and allow a full understanding of the temporal interaction of host and viral genes during baculovirus infection.

A comparison of mRNA extracted from HzSNPV-infected larvae and those of mock-infected larvae revealed a number of differentially regulated genes of physiological and immunological relevance. Induction of the heat shock protein 70 gene (*hsp70*) in response to productive baculovirus infection was previously demonstrated by our lab using 2D-gel electrophoresis analysis and quantitative PCR studies of infected lepidopteran insects and lepidopteran cell lines [26,45]. Our finding of *hsp70* induction in response to baculovirus infection also correlates with recent studies indicating that HSP70 plays an important role in baculovirus expression vector systems [46]. This study shows that virus infection results in the induction of *hsp70* of up to a 177-fold. The heat shock protein family of proteins are well-conserved across the genera and contribute to proper protein folding, organization, and function, especially during stress induced by thermal, chemical, or pathogen-induced cellular perturbations. The hypothesis that baculovirus exploits HSP70 to enhance the availability of properly folded viral proteins during its replication cycle is currently under further study.

In mammals, adhesion modules play a number of roles in response to pathogens, at times serving as a host-defense mechanism, and in other cases they are exploited by pathogens to facilitate dissemination within the host or dispersal without [47–49]. Substantial changes were detected in the expression of cell adhesion molecules *fasciclin-1* and *dystroglycan*, as well as a probable matrix anchor protein, following baculovirus infection. Lepidopteran homologs of *fasciclin-1* and *dystroglycan* have not been well characterized, and what role they play during baculovirus infection remains speculative. Homologs of *fasciclin-2* and *fasciclin-3*, which have roles in neural and midgut development [50–53] were detected in our study, but the level of their respective transcripts was not altered as compared to controls in any of the hemocytes from the challenged insects (data not shown).

*Cecropin D*, an important molecule for bacterial immunity, was also induced in our study by baculovirus infection [54,55]. A suppression of *signal transducing adapter molecule 2* expression of approximately five-fold was noted, although transcription of the signal transducing activator of transcription (*stat*) gene, which plays a critical role in animal immune signaling, was unperturbed (data not shown) in response to any of the treatments.

Fatty acid metabolism and hormone regulation was also altered in response to baculovirus infection. The fatty acid synthase homologs *p260*/*p270* play a role in limb development in *Bombyx mori* [56], and were highly upregulated in response to baculovirus, but not in response to either fungus or bacteria injection. This trend of up-regulation of fatty acid metabolism-related transcripts was generally reflected among the sampled genes, with a number of lipid reductases and desaturases upregulated more than 30-fold following baculovirus infection. Also uniquely reported here, but perhaps not surprisingly, was the magnitude and uniformity of the observed down-regulation of storage proteins *hexamerin*/*arylphorin* in response to baculovirus challenge. Commensurately, transcription of a gene identified as *p82* riboflavin-binding hexamer monomer was suppressed 20-fold in response to baculovirus infection. A role for the extensive modulation of fatty acid metabolism in the replication of baculovirus has yet to be determined, but this apparent shunting of energetic resources from a state of growth to one of infection response has previously been reported in polydnavirus-infected *H. virescens* as well as *Serratia marcescens*-infected honey bees [57,58].

Alterations in transcript levels of genes related to hormonal activity demonstrated the close association between baculovirus infection and the developmental hormones in larvae. A transcript exhibiting homology to the nuclear hormone receptor *HR3*, inducible by ecdysteroid, was upregulated following baculovirus infection. Baculoviruses are known to decrease the ecdysteroid levels in larval hemolymph by glycosylating ecdysteroid through the ecdysteroid UDP-glycosyltransferase (EGT) gene thereby causing larvae to delay pupation [59]. Juvenile hormone (*JH*) epoxide hydrolase and basic *JH* suppressible homologs were inversely regulated: transcription of the former increased between three- and four-fold, while transcription of the latter was suppressed up to 200-fold. Decreasing the JH levels in larvae signals the cessation of feeding and the beginning of the molt process. Alterations or fine tuning of the enzyme levels involved in the *JH* removal would also increase/maintain the *JH* titer and again delay pupation allowing the baculovirus to continue to propagate. The overexpression of *JH* esterase in recombinant baculoviruses has been used to successfully improve their efficacy [60,61].

Errantiviruses have been detected in *Drosophila*, and in lepidopteran species *B. mori* and *Lymantria dispar.* Retroviral sequences were upregulated by baculovirus infection in cultured Sf9 insect cell lines utilized in BEV systems, derived many years ago from *Spodoptera frugiperda*, and also the Hi-5 cell line isolated originally from the cabbage looper *Trichoplusia ni* [62]. Numerous transcripts containing errantivirus and retrovirus-like sequences were detected in our study. It has been observed that insect retroviruses most likely derived portions of their genome from baculoviruses via recombination during their evolution [63]. Menzel and Rohrman speculate a plausible scenario in which baculovirus infection may trigger transposition events, either by a direct mechanism intrinsic to baculovirus(es) or by mitigating the suppressive effect of host silencing factors [62]. Indeed, errantiviruses have been known to “piggy back” by integrating into the baculovirus genome during infection [64], and our report provides further evidence for the activation of errantivirus during productive baculovirus replication in an infected insect.

Overall, this study demonstrates the utility of next-generation sequencing in characterizing host response to various treatments at the transcriptional level. In addition to capturing genetic sequences from a species lacking extensive sequence information, comparison between control and HzSNPV-infected insects has demonstrated key physiological differences in the transcriptional profile of the infected host. Trends were identified that point towards a decrease in host energy storage, alterations in transcription of hormonal genes affecting molting and pupation, altered transcription of genes related to cell adhesion and motility, and a generalized stress response that resulted in the virus-specific transcriptional induction of discrete heat shock response constituents. Analysis of baculovirus-specific transcripts has revealed the coordinated expression of key viral genes following infection, as well as a surprising number of late genes that appeared to be transcribed earlier than expected. Further, assembly of the contigs isolated from the hemocytes of baculovirus-infected insects netted a number of differentially regulated retroviral-transcripts that were deduced to be errantiviruses, of which very little is currently known. Further mining of these data and subsequent experimentation is expected to add to the depth and breadth of our understanding of the complex interactions that govern the outcome of baculovirus infection in their lepidopteran hosts.

## Figures and Tables

**Table 1.** Quantity of hemocyte mRNA sequences obtained from control and treated groups.

**Treatment**	**Total Seqs**	**After Cleaning**	**After Decontamination**	**Aligned**	**Uniquely Aligned**
Control	50396515	48734654	48646727	31308740	19809154
Bacterial	53593477	51046869	51000867	32021434	21516663
Fungal	44558357	42149252	42139873	25948555	17600091
Viral	53944832	50600536	50575131	32107380	20716956

**Table 2.** Illumina sequence reads that match more than one HzSNPV ORF.

**HzSNPV ORF**	**HzSNPV ORF Name**	**Corresponding AcMNPV ORF**	**ORF Start**	**ORF End**	**# of Hits Unique to ORF**	**# of Hits ORF**	**Difference in Hits**
26	ORF26		23950	24102	22	29	7
27	ORF27	34	24045	24812	459	1256	797
28	ubiquitin	35	24652	24903	600	1390	790
46	ORF46		39876	40103	616	618	2
47	lef-10	53a	40063	40278	298	676	378
48	vp1054	54	40151	41206	3032	3408	376
75	gp41	80	65470	66438	5648	5791	143
76	ORF76	81	66368	67093	1488	2135	647
77	ORF77	82	66966	67643	1146	1669	523
78	vp91capsid	83	67573	70023	140	159	19
86	viral_ppase	98	80264	81229	1185	1361	176
87	lef-5	99	81125	82072	6877	7053	176
127	ORF127		119843	120421	481	489	8
128	BV-ec31	17	120372	121172	1754	1762	8

**Table 3.** Differential regulation of stress response, adhesion, and immunity-related genes.

**Function/Gene ID**	**Control**	**NPV**	**Fold Change**	**Length**	**%Cov**	**e-value**
**Heat shock**						
Heat shock protein 70 *Helicoverpa zea*	24	4238	176.6	1452	69	0
Heat shock protein 70 *Spodoptera exigua*	30	2122	70.7	1763	98	0
PREDICTED: similar to HSP70 *Acyrthosiphon pisum*	1	85	85.0	170	100	6E-05
Hsp70-interacting protein *H. armigera*	23	1934	84.1	599	94	2E-126
Heat shock protein 68 *Drosophila yakuba*	1	72	72.0	150	100	1E-39
Heat shock protein 19.5 *Sesamia nonagrioides*	3	64	21.3	171	100	7E-13
Heat shock protein hsp20.4 *Bombyx mori*	1	21	21.0	200	100	2E-08
Heat shock protein 19.7 *Mamestra brassicae*	1	19	19.0	87	100	4E-12
Heat shock protein 20.8 *Bombyx mori*	5	80	16.0	105	100	5 E-45

**Cell action**						
EGF-containing fibulin-like ECM prot. 2 *Macaca fascicularis*	65	462	7.1	2092	100	2E-64
Cecropin D *Spodoptera litura*	1	12	12.0	263	64	1E-12
Larval cuticle protein *Bombyx mori*	4	26	6.5	163	100	5E-25
Dystroglycan *Bos taurus*	5990	1575	0.3	2924	60	2E-44
Signal transducing adapter molecule 2 *Rattus norvegicus*	420	88	0.2	417	100	3E-13
Fasciclin-1 *Schistocerca americana*	1383	23	0.02	755	72	1E-103

NPV: HzSNPV infected samples; %Cov: percent coverage; e-value: expect value.

**Table 4.** Differentially regulated metabolism, storage, and hormone-related genes.

**Function/Gene ID**	**Control**	**NPV**	**Fold Change**	**Length**	**%Cov**	**e-value**
**Hormone**						
Probable nuclear hormone receptor HR3 *Manduca sexta*	33	253	7.7	99	100	7E-34
Nuclear hormone receptor FTZ-F1 *Bombyx mori*	766	3232	4.2	2867	98	0
Juvenile hormone epoxide hydrolase *Helicoverpa armigera*	25	89	3.6	657	100	3E-22
Hormone receptor 3C *Choristoneura fumiferana*	5	15	3.0	124	100	3E-13
Basic juvenile hormone-suppressible protein 1 *Trichoplusia ni*	2575	67	0.03	2279	78	0
Juvenile hormone diol kinase *Manduca sexta*	58	11	0.2	567	100	4E-59
Basic juvenile hormone-suppressible protein 2 *Trichoplusia ni*	199	1	0.01	2170	40	0

**Lipid Metabolism**						
P260 *Bombyx mori*	15	6420	428.0	4896	92	0
Stearoyl-CoA desaturase *Aedes aegypti*	1	237	237.0	775	87	1E-74
Fatty-acyl CoA reductase *Ostrinia nubilalis*	5	1038	207.6	1520	88	2E-179
Fatty acid synthase (Fragment) *Heliothis virescens*	2	149	74.5	548	93	8E-100
Acyl-CoA delta-9 desaturase isoform *Helicoverpa zea*	19	1922	101.16	1238	94	7E-131
P270 *Bombyx mori*	6	306	51.0	959	81	2E-133
Fatty acyl-CoA desaturase *Glossina morsitans*	1	38	38.0	190	100	6E-16
Acyl-CoA desaturase (Fragment) *Heliothis virescens*	0	36	36.0	215	100	2E-37
Acyl-CoA-delta-6-desaturase *Antheraea pernyi*	0	19	19.0	141	100	2E-12
Acyl-CoA oxidase (Fragment) *H. virescens*	0	25	25.0	295	100	2E-29
PREDICTED: similar to acyl-CoA oxidase A*cyrthosiphon pisum*	0	24	24.0	145	61	6E-21
Peroxisomal acyl-CoA oxidase, putative Ixodes scapularis	1	29	29.0	252	96	4E-24
Acyl-CoA synthetase short-chain family member 3, mitochondrial *Camponotus floridanus*	0	16	16.0	159	89	7E-22
Acyl-CoA desaturase PintNPRD (Fragment) *Plodia interpunctella*	0	12	12.0	158	56	5E-24
Lipid storage droplets surface-binding protein *Drosophila melanogaster*	22	70	3.2	494	85	2E-31
Fatty acid beta-oxidation complex subunit beta *Heliothis virescens*	11	34	3.1	204	92	2E-12
Glycine C-acetyltransferase/2-amino-3-ketobutyrate-CoA ligase *Ixodes scapularis*	45	11	0.2	185	100	2E-21
Fatty acid-binding protein 3 *Helicoverpa armigera*	25	5	0.2	65	100	1E-11
Probable malonyl-CoA-acyl carrier protein transacylase *Drosophila melanogaster*	171	23	0.13	272	100	1E-14
Low-density lipoprotein receptor-related protein 1 *Gallus gallus*	45	3	0.07	97	100	2E-9

**Storage**						
Arylphorin subunit *Spodoptera litura*	2646	172	0.07	1324	84	0
Hexamerine *Helicoverpa armigera*	100	0	0.01	210	100	4E-145
P82 riboflavin-binding hexamer *Heliothis virescens*	19	1	0.05	353	93	2E-13

NPV: HzSNPV infected samples; %Cov: percent coverage; e-value: expect value.

**Table 5.** Differentially regulated errantivirus (retrovirus-like) genes.

**Gene ID**	**Control**	**NPV**	**Fold Change**	**Length**	**%Cov**	**e-value**
Endonuclease-reverse transcriptase *Bombyx mori*	4	45	11.2	260	100	3E-23
RNase H and integrase-like protein (Fragment) *Bombyx mori*	6	60	10.0	162	100	4E-6
PREDICTED: similar to Copia protein (Gag-int-pol protein) *Tribolium castaneum*	49	402	8.2	1164	97	1E-101
PREDICTED: similar to putative gag-pol protein, partial *Acyrthosiphon pisum*	6	48	8.0	589	100	1E-35
RNA-directed DNA polymerase (Reverse transcriptase) *Medicago truncatula*	60	366	6.1	980	100	1E-88
Protease and reverse transcriptase-like Protein *Bombyx Mori*	65	386	5.9	928	100	3E-86
Putative gag-pol polyprotein *Aster yellows phytoplasma*	36	202	5.6	939	100	2E-43
Gag-like protein *Bombyx mori*	68	368	5.4	799	89	2E-61
PREDICTED: similar to protease, reverse transcriptase, ribonuclease H, integrase *Tribolium castaneum*	11	49	4.5	113	100	5E-23
PREDICTED: similar to gag-pol polyprotein *Tribolium Castaneum*	75	304	4.1	1025	100	6E-35
PREDICTED: similar to protease, reverse transcriptase, ribonuclease H, integrase *Tribolium castaneum*	27	103	3.8	307	78	1E-18
Enzymatic polyprotein; Endonuclease; Reverse transcriptase, putative *Pediculus humanus corporis*	15	55	3.7	355	100	1E-19
Putative uncharacterized protein (Gag-pol polyprotein) *Drosophila melanogaster*	26	87	3.4	765	80	6E-52
PREDICTED: similar to Copia protein (Gag-int-pol protein) *Tribolium castaneum*	110	0	0.01	448	100	2E-31

NPV, HzSNPV infected samples; %Cov, percent coverage; e-value, expect value.

## References

[1] Grabherr R., Ernst W. (2010). Baculovirus for eukaryotic protein display. Curr. Gene Ther.

[2] Liu C.Y., Chen H.Z., Chao Y.C. (2010). Maximizing baculovirus-mediated foreign proteins expression in mammalian cells. Curr. Gene Ther.

[3] Passarelli A.L., Guarino L.A. (2007). Baculovirus late and very late gene regulation. Curr. Drug Targets.

[4] Szewczyk B., Hoyos-Carvajal L., Paluszek M., Skrzecz I., Lobo de S.M. (2006). Baculoviruses—Re-emerging biopesticides. Biotechnol. Adv.

[5] Granados R.R., Lawler K.A., Burand J.P. (1981). Replication of *Heliothis zea* baculovirus in an insect cell line. Intervirology.

[6] McIntosh A.H., Ignoffo C.M., Andrews P.L. (1985). *In vitro* host range of five baculoviruses in lepidopteran cell lines. Intervirology.

[7] Washburn J.O., Wong J.F., Volkman L.E. (2001). Comparative pathogenesis of *Helicoverpa zea* S nucleopolyhedrovirus in noctuid larvae. J. Gen. Virol.

[8] Werner T. (2011). Next generation sequencing allows deeper analysis and understanding of genomes and transcriptomes including aspects to fertility. Reprod. Fertil. Dev.

[9] Alkan C., Coe B.P., Eichler E.E. (2011). Genome structural variation discovery and genotyping. Nat. Rev. Genet.

[10] Lam C.W., Lau K.C., Tong S.F. (2010). Microarrays for personalized genomic medicine. Adv. Clin. Chem.

[11] Yao Q., Li M.W., Wang Y., Wang W.B., Lu J., Dong Y., Chen K.P. (2003). Screening of molecular markers for NPV resistance in *Bombyx mori* L. (Lep., Bombycidae). J. Appl .Entomol.

[12] Kidokoro K., Ito K., Ogoyi D.O., Abe H., Mita K. (2010). Non-susceptibility genes to *Bombyx* densovirus type 1, Nid-1 and nsd-1, affect distinct steps of the viral infection pathway. J. Invertebr. Pathol.

[13] Asser-Kaiser S., Radtke P., El-Salamouny S., Winstanley D., Jehle J.A. (2011). Baculovirus resistance in codling moth (*Cydia pomonella* L.) caused by early block of virus replication. Virology.

[14] Sessions O.M., Barrows N.J., Souza-Neto J.A., Robinson T.J., Hershey C.L., Rodgers M.A., Ramirez J.L., Dimoupoulos G., Yang P.L., Pearson J.L., Garcia-Blanco M.A. (2009). Discovery of insect and human dengue virus host factors. Nature.

[15] Wang J.H., Vallene S., Ramet M. (2010). *Drosophila* as a model for antiviral immunity. World J. Biol. Chem.

[16] Sabin L.R., Hanna S.L., Cherry S. (2010). Innate antiviral immunity in *Drosophila*. Curr. Opin. Immunol.

[17] Hao L., Sakurai A., Watanabe T., Sorensen E., Nidom C.A., Newton M.A., Ahlquist P., Kawaoka Y. (2008). *Drosophila* RNAi screen identifies host genes important for influenza virus replication. Nature.

[18] Steinert S., Levashina A.A. (2011). Intracellular immune responses of dipteran insects. Immunol. Revs.

[19] Girard Y.A., Mayhew G.F., Fuchs J.F., Li H., Schneider B.S., McGee C.E., Rocheleau T.A., Helmy H., Christensen B.M., Higgs S., Bartholomay L.C. (2010). Transcriptome changes in *Culex quinquefasciatus* (Diptera: Culicidae) salivary glands during West Nile virus infection. J. Med. Entomol.

[20] Blair C.D. (2011). Mosquito RNAi is the major innate immune pathway controlling arbovirus infection and transmission. Future Microbiol.

[21] Yang Z., Bruno D.P., Martens C.A., Porcella S.F., Moss B. (2010). Simultaneous high-resolution analysis of vaccinia virus and host cell transcriptomes by deep RNA sequencing. Proc. Natl. Acad. Sci. U. S. A.

[22] Bengali Z., Satheshkumar P.S., Yang Z., Weisberg A.S., Paran N., Moss B. (2011). *Drosophila* S2 cells are non-permissive for vaccinia virus DNA replication following entry via low pH-dependent endocytosis and early transcription. PLoS One.

[23] Clem R.J., Popham H.J.R., Shelby K.S., Asgari S., Johnson K. (2011). Antiviral respnse in insects: Apoptosis and humoral responses. Insect Virology.

[24] Whitfield A.E., Rotenberg D., Aritua V., Hogenhout S.A. (2011). Analysis of expressed sequence tags from Maize mosaic rhabdovirus-infected gut tissues of *Peregrinus maidis* reveals the presence of key components of insect innate immunity. Insect Mol. Biol.

[25] Luan J.-B., Li J.-M., Varela N., Wang Y.-L., Li F.-F., Bao Y.-Y., Zhang C.-X., Liu S.-S., Wang X.-W. (2011). Global analysis of the transcriptional response of the whitefly to *Tomato Yellow Leaf Curl China Virus* reveals the realtionship of coevolved adaptations. J. Virol.

[26] Popham H.J.R., Grasela J.J., Goodman C.L., McIntosh A.H. (2010). Baculovirus infection influences host protein expression in two established insect cell lines. J. Insect Physiol.

[27] Chen X., Zhang W.J., Wong J., Chun G., Lu A., McCutchen B.F., Presnail J.K., Herrmann R., Dolan M., Tingey S. (2002). Comparative analysis of the complete genome sequences of *Helicoverpa zea* and *Helicoverpa armigera* single-nucleocapsid nucleopolyhedroviruses. J. Gen. Virol.

[28] Ayres M., Howard S.C., Kuzio J., Lopez-Ferber M., Possee R.D. (1994). The complete DNA sequence of *Autographa californica* nuclear polyhedrosis virus. Virology.

[29] Ludwig A., Valente V.L., Loreto E.L. (2008). Multiple invasions of Errantivirus in the genus Drosophila. Insect Mol. Biol.

[30] Popham H.J.R., Shelby K.S., Popham T.W. (2005). Effect of dietary selenium supplementation on resistance to baculovirus infection. Biol. Contr.

[31] Terenius O., Popham H.J.R., Shelby K.S. (2009). Bacterial, but not baculoviral infections stimulate Hemolin expression in noctuid moths. Dev. Comp. Immunol.

[32] Shelby K.S., Popham H.J.R. (2006). Plasma phenoloxidase of larval *Heliothis virescens* is virucidal. J. Insect Sci.

[33] Shelby K.S., Popham H.J.R. (2009). Analysis of ESTs generated from immune-stimulated hemocytes of larval *Heliothis virescens*. J. Invertebr. Pathol.

[34] Marioni J.C., Mason C.E., Mane S.M., Stephens M., Gilad Y. (2008). RNA-seq: An assessment of technical reproducibility and comparison with gene expression arrays. Genome Res.

[35] FASTX-Tool Kit FASTQ/A Short-Reads Pre-Processing Tools. hannonlab.cshl.edu/fastx_toolkit/.

[36] Zerbino D.R., Birney E. (2008). Velvet: Algorithms for *de novo* short read assembly using de Bruijn graphs. Genome Res.

[37] Shulz M., Zerbino D. Oases *De novo* assembler for very short reads. http://www.ebi.ac.uk/~zerbino/oases/j/.

[38] Kurtz S. The Vmatch large scale sequence analysis software. http://www.vmatch.de/.

[39] Jain E., Bairoch A., Duvaud S., Phan I., Redaschi N., Suzek B.E., Martin M.J., McGarvey P., Gasteiger E. (2009). Infrastructure for the life sciences: Design and implementation of the UniProt website. BMC Bioinformatics.

[40] Conesa A., Götz S., García-Gómez J.M., Terol J., Talón M., Robles M. (2005). Blast2GO: A universal tool for annotation, visualization and analysis in functional genomics research. Bioinformatics.

[41] *Heliothis virescens* Transcriptome Project. http://www.ncbi.nlm.nih.gov/bioproject/49697/.

[42] Li R., Li Y., Kristiansen K., Wang J. (2008). SOAP: Short oligonucleotide alignment program. Bioinformatics.

[43] Washburn J.O., Wong J.F., Volkman L.E. (2001). Comparative pathogenesis of Helicoverpa zea S nucleopolyhedrovirus in noctuid larvae. J. Gen. Virol.

[44] Washburn J.O., Trudeau D., Wong J.F., Volkman L.E. (2003). Early pathogenesis of *Autographa californica* multiple nucleopolyhedrovirus and *Helicoverpa zea* single nucleopolyhedrovirus in *Heliothis virescens*: A comparison of the ‘M’ and ‘S’ strategies for establishing fatal infection. J. Gen. Virol.

[45] Breitenbach J.E., Popham H.J.R. (2011). Baculovirus infection induces heat shock response *in vitro* and *in vivo*.

[46] Lyupina Y.V., Dmitrieva S.B., Timokhova A.V., Beljelarskaya S.N., Zatsepina O.G., Evgen’ev M.B., Mikhailov V.S. (2010). An important role of the heat shock response in infected cells for replication of baculoviruses. Virology.

[47] Dustin M. (2003). Viral spread through protoplasmic kiss. Nat. Cell Biol.

[48] Hutt-Fletcher L.M., Chesnokova L.S. (2010). Integrins as triggers of Epstein-Barr virus fusion and epithelial cell infection. Virulence.

[49] Thomsen A.R., Nansen A., Madsen A.N., Bartholdy C., Christensen J.P. (2003). Regulation of T cell migration during viral infection: role of adhesion molecules and chemokines. Immunol. Lett.

[50] Nardi J.B., Bee C.M., Miller L.A., Mathur D., Ohlstein B. (2011). Cell renewal in adjoining intestinal and tracheal epithelia of Mandu*ca*. J. Insect Physiol..

[51] Hebbar S., Fernandes J.J. (2010). Glial remodeling during metamorphosis influences the stabilization of motor neuron branches in *Drosophila*. Dev. Biol.

[52] Mao Y., Freeman M. (2009). Fasciclin 2, the *Drosophila* orthologue of neural cell-adhesion molecule, inhibits EGF receptor signalling. Development.

[53] Nassif C., Noveen A., Hartenstein V. (2003). Early development of the Drosophila brain: III. The pattern of neuropile founder tracts during the larval period. J. Comp Neurol.

[54] Lemaitre B. (2004). The road to toll. Nat. Rev. Immunol.

[55] Dimopoulos G., Muller H.M., Levashina E.A., Kafatos F.C. (2001). Innate immune defense against malaria infection in the mosquito. Curr. Opin. Immunol.

[56] Ueno K., Suzuki Y. (1997). p260/270 expressed in embryonic abdominal leg cells of *Bombyx mori* can transfer palmitate to peptides. J. Biol. Chem.

[57] Lourenco A.P., Martins J.R., Bitondi M.M., Simoes Z.L. (2009). Trade-off between immune stimulation and expression of storage protein genes. Arch. Insect Biochem. Physiol.

[58] Shelby K.S., Webb B.A. (1994). Polydnavirus infection inhibits synthesis of an insect plasma protein, arylphorin. J. Gen. Virol.

[59] O’Reilly D.R. (1995). Baculovirus-encoded ecdysteroid UDP-glucosyltransferases. Insect Biochem. Mol. Biol.

[60] Bonning B.C., Ward V.K., van Meer M.M., Booth T.F., Hammock B.D. (1997). Disruption of lysosomal targeting is associated with insecticidal potency of juvenile hormone esterase. Proc. Natl. Acad. Sci. U. S. A.

[61] Wroblewski V.J., Harshman L.G., Hanzlik T.N., Hammock B.D. (1990). Regulation of juvenile hormone esterase gene expression in the tobacco budworm (*Heliothis virescens*). Arch. Biochem. Biophys.

[62] Menzel T., Rohrmann G.F. (2008). Diversity of errantivirus (retrovirus) sequences in two cell lines used for baculovirus expression, *Spodoptera frugiperda* and *Trichoplusia ni*. Virus Genes.

[63] Pearson M.N., Rohrmann G.F. (2006). Envelope gene capture and insect retrovirus evolution: the relationship between errantivirus and baculovirus envelope proteins. Virus Res.

[64] Miller D.W., Miller L.K. (1982). A virus mutant with an insertion of a copia-like transposable element. Nature.

